# Games for Girls

**Published:** 1921-06-25

**Authors:** 


### Games for Girls.

With a recent issue of *The Teacher's World* is published an altogether admirable supplement dealing entirely with school games. Special interest attaches to an article on "The Best Games for Girls," by Miss Elizabeth C. Terry, organiser of physical training to the Lindsey Education Committee, because of the controversy recently started as to whether the athletic inclinations of the modern English girl are dangerous to ill-health. Miss Terry refrains, perhaps wisely, from joining directly in this so far not very conclusive discussion; but in her interesting "scheme of play" and in her analysis of the various games that are played or may be played in girls' schools she completely bears out the argument previously urged in these columns that the suitability or unsuitability of organised games depends not so much on the games as on the skill and judgment of the organiser and on the watchful supervision of the games mistress. It is instructive to note that Miss Terry takes cricket for granted, as a game "whose merits are well known," though she judiciously adds that if it is to be played to any purpose it requires a level ground "and skilled coaching." With regard to hockey she is inclined to be cautious. Though it "provides splendid exercise for the majority of those taking part in the game" and "cultivates quick hand and eye work," besides arousing very strongly the team spirit, she thinks that it is too strenuous for the average schoolgirl and is in danger of causing overstrain, especially when it is played, as is so often the case, only in one afternoon a week, so that the child is not in training. That is perfectly true, but here again it is a question of proper organisation. Miss Terry, we think, makes too much of the "additional disadvantage" that it encourages a stooping position and, by exercising one side of the body more than the other, tends to the development of minor degrees of spinal curvature. Most people who have actually played hockey will agree that in practice this is not the case, for the straight sprinting that the game demands more than counterbalances any slight tendency to one-sidedness through holding the stick on the right side. Theoretically one might make the same criticism of cricket—certainly in batting, and less definitely in bowling. "Among players," she adds, "with no knowledge of how games should be played it often becomes far too rough, and sometimes dangerous, *unless* [the italics are the lady's] it is adequately supervised by an experienced and skilful instructor." Precisely. We should like to italicise the italics. Skilful supervision is the crux of the matter.

